# Increased Secreted Amyloid Precursor Protein-α (sAPPα) in Severe Autism: Proposal of a Specific, Anabolic Pathway and Putative Biomarker

**DOI:** 10.1371/journal.pone.0020405

**Published:** 2011-06-22

**Authors:** Balmiki Ray, Justin M. Long, Deborah K. Sokol, Debomoy K. Lahiri

**Affiliations:** 1 Department of Psychiatry, Institute of Psychiatric Research, Indiana University School of Medicine, Indianapolis, Indiana, United States of America; 2 Department of Neurology, Indiana University School of Medicine, Indianapolis, Indiana, United States of America; 3 Department of Medical and Molecular Genetics, Indiana University School of Medicine, Indianapolis, Indiana, United States of America; Mental Health Research Institute of Victoria, Australia

## Abstract

Autism is a neurodevelopmental disorder characterized by deficits in verbal communication, social interactions, and the presence of repetitive, stereotyped and compulsive behaviors. Excessive early brain growth is found commonly in some patients and may contribute to disease phenotype. Reports of increased levels of brain-derived neurotrophic factor (BDNF) and other neurotrophic-like factors in autistic neonates suggest that enhanced anabolic activity in CNS mediates this overgrowth effect. We have shown previously that in a subset of patients with severe autism and aggression, plasma levels of the secreted amyloid-β (Aβ) precursor protein-alpha form (sAPPα) were significantly elevated relative to controls and patients with mild-to-moderate autism. Here we further tested the hypothesis that levels of sAPPα and sAPPβ (proteolytic cleavage products of APP by α- and β-secretase, respectively) are deranged in autism and may contribute to an anabolic environment leading to brain overgrowth. We measured plasma levels of sAPPα, sAPPβ, Aβ peptides and BDNF by corresponding ELISA in a well characterized set of subjects. We included for analysis 18 control, 6 mild-to-moderate, and 15 severely autistic patient plasma samples. We have observed that sAPPα levels are increased and BDNF levels decreased in the plasma of patients with severe autism as compared to controls. Further, we show that Aβ1-40, Aβ1-42, and sAPPβ levels are significantly decreased in the plasma of patients with severe autism. These findings do not extend to patients with mild-to-moderate autism, providing a biochemical correlate of phenotypic severity. Taken together, this study provides evidence that sAPPα levels are generally elevated in severe autism and suggests that these patients may have aberrant non-amyloidogenic processing of APP.

## Introduction

Autism, which is a neurodevelopmental disorder, is mostly characterized by deficits in verbal communication, impairment in social interactions, and the presence of repetitive, stereotyped, and compulsive behaviors. [1]. First described by Kanner in 1943 [2], the prevalence of the disorder has increased in recent years to near 1 in 110 [3], [4], [5], likely a combination of increased social awareness, broader disease classification, and true increase in disorder prevalence. Unfortunately, the neurobiological basis of the disorder is at present very poorly understood. Behavioral phenotypes exhibit very high heterogeneity, which has led to the concept of the autism spectrum. Studies of twins show high autism spectrum concordance between monozygotic twins (60–90%) and very low concordance between dizygotic twins (10%), stressing the significant heritable basis of the disorder [6]. Syndromes with increased prevalence of autism (i.e. Fragile X syndrome, Rett syndrome) and rare mutations linked to the disorder have highlighted the contribution of synaptic dysfunction to disorder etiology [7].

A widely replicated finding made by both head circumference measurements and volumetric MRI is the presence of macrocephaly in a proportion of autistic patients (15–30%), with up to 90% demonstrating some degree of brain overgrowth in early years [8], [9], [10], [11]. This trajectory of early brain overgrowth has also been established in at least one longitudinal study [12]. The exact nature of this overgrowth is not quite clear, as expected increases in neuronal and synaptic densities were not found when autistic brains were analyzed by N-acetylaspartate magnetic resonance spectroscopy. Instead reduced neuronal and synaptic densities were found [13]. However, studies have found increased levels of brain-derived neurotrophic factor (BDNF) and other neurotrophic factors in the blood of autistic neonates and adults [14], [15], [16], suggesting that enhanced anabolic activity in the CNS may mediate this overgrowth effect.

In this context, we previously assayed the peripheral levels of the secreted alpha-secretase product of the amyloid-β precursor protein (APP), or sAPPα, in autistic and control samples [17]. APP is a type I transmembrane protein that undergoes proteolytic processing by secretase enzymes to liberate soluble fragments. APP expression is regulated at both the transcriptional and post-transcriptional level. The APP promoter has a complex structure containing many proximal and distal regulatory elements that mediate constitutive and stimulated regulatory activities [18], [19], [20], [21], [22], [23]. Regulatory elements in the 5′UTR can independently drive promoter activity, such as a reported CAGA box [24]. Regulatory elements in the 5′UTR may also post-transcriptionally regulate APP expression. Examples include an iron responsive element [25] and IL-1 responsive element [26] involved in regulating stimulated translation of the APP transcript. The APP 3′UTR also mediates post-transcriptional regulation through several stability control elements that regulate APP mRNA stability [27], [28], [29]. MicroRNA sites in the 3′-UTR also regulate APP expression, with miR-101 mediating a very potent inhibitory effect on translation [30], [31]. Once expressed, APP is sequentially cleaved by β-secretase (BACE1) and the γ-secretase complex, releasing sAPPβ and amyloid-β (Aβ) peptide, the major component of amyloid plaques found in Alzheimer's disease [32]. Aβ is liberated from APP in two predominant forms: the more abundant 40 amino acid form (Aβ1-40) and the less abundant, more fibrillogenic, 42 amino acid form (Aβ1-42). Alternative cleavage of APP by α-secretase and γ-secretase releases the non-amyloidogenic 3kDa peptide (p3 peptide) and sAPPα, instead of intact 4 kDa Aβ and sAPPβ [32], [33]. sAPPγ may be released if APP is cleaved by the γ-secretase complex at the C-terminus of the Aβ domain prior to α- or β-secretase cleavage. The various forms of sAPP are distinguished by the length of Aβ domain contained at the C-terminal end: sAPPγ contains the full Aβ domain, sAPPα contains only residues 1–6 of Aβ, and sAPPβ completely lacks the Aβ sequence. These three sAPP species primarily constitute the pool of total sAPP in the human plasma as mentioned subsequently in the text. Notably, sAPPα has been well characterized and previously found to exhibit a wide array of neurotrophic activities [34], [35] that might be important for neurodevelopment. Further, sAPPα has been shown to stimulate the transdifferentiation of adult bone marrow progenitor cells (MAPCs) to neuronal phenotypes [36].

We postulate that elevated levels of sAPPα could also contribute to brain overgrowth in a pathological state. In our previous study, we discovered that in a subset of patients with severe autism and aggression, plasma levels of sAPPα were significantly elevated relative to controls and patients with mild-to-moderate autism [17]. This has led us to the hypothesis that sAPPα may contribute to an anabolic environment in the central nervous system (CNS) leading to brain overgrowth in autism. However, one weakness of the previous study was small sample size. Here, we extend our previous finding of increased sAPPα levels in severe autism in a larger, independent cohort of patients. For the first time we demonstrate that the increase in the sAPPα level is a general finding in severe autism that does not require an aggressive behavioral phenotype. Further, we find that BDNF levels are decreased in the plasma of patients with severe autism as compared to controls. Crucially, we also report a decrease in levels of both Aβ1-40 and Aβ1-42 peptides and sAPPβ in severely autistic patients as compared to controls. These results suggest that the non-amyloidogenic processing pathway may be favored in severe autism with the implication that increased neurotrophic sAPPα may contribute to a state of anabolic excess promoting overgrowth in the autistic brain.

## Results

### Sample Demographics

Thirty-nine patients, including 34 males, were recruited for this study using the inclusion criteria described above (Table 1). Eighteen patients with no overt neurological or psychiatric abnormalities served as normal controls. Six patients with confirmed autism diagnoses were classified with mild-to-moderate disease as specified by CARS score between 30 and 36.5. Fifteen autistic patients were classified as severe based on CARS score of 37 or greater. Mean ages (in years ±SD) at the time of plasma collection for controls, mild-to-moderate and severely autistic patients were 8.17±4.33, 6.16±0.68, and 6.40±3.04, respectively. One-way ANOVA did not detect any statistically significant differences in the mean age between any of the groups (F = 1.316, p = 0.281). Two children with autism had epilepsy. All children with severe autism were mentally retarded as determined by IQ scores less than 70. Medication history and other clinical features for most patients were non-significant.

**Table 1:** Demographic information for study participants.

*Patient No*	*Dx*	*CARS*	*Age (yrs)^a^*	*Sex*	*Ethnicity*
1	Normal	15	7.7	Male	Caucasian
2	Normal	15	6.0	Male	Caucasian
3	Normal	15	5.3	Female	Caucasian
4	Normal	15	4.8	Female	Caucasian
5	Normal	15	6.5	Male	Caucasian
6	Normal	15	4.2	Female	Caucasian
7	Normal	15	7.7	Male	Hispanic
8	Normal	15	11.8	Male	Caucasian
9	Normal	15	14.7	Male	Caucasian
10	Normal	15	18.3	Male	Caucasian
11	Normal	18	11.3	Male	Caucasian
12	Normal	15	9.6	Male	Caucasian
13	Normal	15	13.6	Male	Caucasian
14	Normal	15	3.8	Male	Caucasian
15	Normal	15	7.5	Male	Caucasian
16	Normal	15	2.7	Male	Caucasian
17	Normal	15	2.8	Male	Caucasian
18	Normal	15	8.9	Male	Caucasian
19	Autism (M)	30	5.7	Male	Caucasian
20	Autism (M)	33	7.0	Male	Caucasian
21	Autism (M)	36	6.4	Male	Caucasian
22	Autism (M)	30	6.5	Male	Caucasian
23	Autism (M)	33	6.3	Male	Caucasian
24	Autism (M)	32.5	5.1	Male	Caucasian
25	Autism (S)	50	6.0	Male	Caucasian
26	Autism (S)	52	5.5	Male	Caucasian
27	Autism (S)	42	6.7	Male	Caucasian
28	Autism (S)	57	6.4	Male	Caucasian
29	Autism (S)	48	5.9	Male	Caucasian
30	Autism (S)	43	5.2	Male	Caucasian
31	Autism (S)	47	5.6	Male	Caucasian
32	Autism (S)	37	4.7	Female	Caucasian
33	Autism (S)	43	7.8	Female	Caucasian
34	Autism (S)	39	7.9	Male	Caucasian
35	Autism (S)	39	2.8	Male	Caucasian
36	Autism (S)	40	5.9	Male	Caucasian
37	Autism (S)	56	6.1	Male	Caucasian
38	Autism (S)	45	3.2	Male	Caucasian
39	Autism (S)	53	16.5	Male	Caucasian

(M), mild-to-moderate autism; (S), severe autism; CARS, Childhood Autism Rating Scale total scores.

*^a^*Age in years at time of blood draw.

### sAPPα is Elevated in the Plasma of Severely Autistic Patients

**Figure 1:**
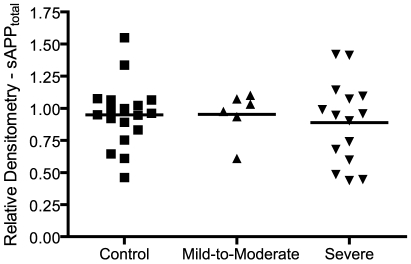
Plasma levels of total sAPP in control and autistic patients. Plasma levels of total sAPP (α and β) were assayed by Western blot from control and autistic patients. Autistic patients were classified as mild-to-moderate or severe based on clinical CARS score. Total sAPP bands were analyzed by scanning the final blot followed by densitometry, ImageJ quantification, and normalization against β-actin bands. Statistical significance was assessed using ANOVA. No significant differences observed in mean total sAPP levels between control or autistic patients.

As a first step in characterizing the expanded set of patient plasma samples encompassing control, mild-to-moderate, and severely autistic patients, plasma levels of total sAPP (α and β combined) were analyzed by Western blot using the well-characterized monoclonal anti-N-terminus APP antibody 22C11 [45] , which detects C-terminal truncated sAPP in the absence of full-length APP. HSA-immunosubtracted plasma samples were used in this analysis. Total sAPP bands were densitometrically quantified using Image J software. No significant difference was observed between control and either category of autistic patient by ANOVA (F = 0.239, p = 0.788) (Figure 1).

**Figure 2:**
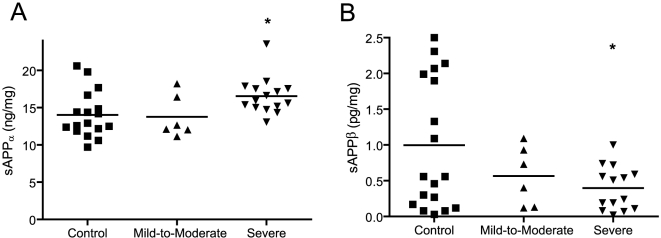
Plasma levels of sAPPα and sAPPβ in control and autistic patients. (A) Plasma sAPPα and (B) sAPPβ levels were quantified from albumin-depleted samples of control and autistic patients by corresponding ELISA procedures. sAPPα and sAPPβ quantities were normalized against immunodepleted plasma protein concentrations. No difference was observed in the mean plasma sAPPα and sAPPβ levels of mild-to-moderate cases of autism relative to controls. Plasma sAPPα levels were significantly increased and sAPPβ levels decreased in severely autistic patients relative to controls Statistical significance assessed using ANOVA followed by post-hoc Dunnett's t-test for multiple comparisons (*p<0.05).

To determine if the primary biochemical change observed in our previous study of autistic and control patients was also observed in the larger patient set under study here, sAPPα levels were specifically assayed by ELISA. Initial tests revealed that the linearity and sensitivity of this ELISA was suboptimal due to non-specific binding attributed to the high protein content of unmodified human plasma (data not shown). Therefore, all plasma samples were HSA-immunosubtracted prior to analysis. No change was observed in the mean levels of sAPPα between control and mild-to-moderate autistic patients. Notably, a very distinct and statistically significant increase in sAPPα (∼20%) was observed in severely autistic patients relative to controls following ANOVA (F = 3.82, p = 0.032) and post-hoc Dunnett's multiple comparison test (p = 0.032) (Figure 2). This confirms the finding of increased plasma sAPPα in severely autistic patients with aggression described in our previous study but, importantly, the results described here did not require coexisting behavioral aggression. Therefore, elevated peripheral sAPPα appears to be a trend observed in the broader population of severely autistic patients. sAPPα ELISA levels were further normalized to total sAPP Western signals (relative units) and while there was a trend for elevated sAPPα:total sAPP ratios in severely autistic samples (data not shown), no statistically significant differences were observed between any groups following ANOVA (F = 2.725, p = 0.079).

### sAPPβ is Decreased in the Plasma of Severely Autistic Patients

To determine if the secreted APP product of amyloidogenic processing, sAPPβ, was altered in the plasma of autistic patients in this plasma set, sAPPβ levels were assayed in HSA-immunosubtracted plasma by a specific sandwich ELISA. Indeed, sAPPβ levels were significantly decreased (∼60%) in the plasma of severely autistic patients relative to controls (Figure 2B) as determined by ANOVA (F = 3.223, p = 0.05) and post-hoc Dunnett's multiple comparison test (p = 0.035). Ratios of sAPPβ:total sAPP were also calculated and no significant differences were observed between any groups (data not shown) following ANOVA (F = 1.876, p = 0.168).

### Aβ Peptides are Decreased in the Plasma of Severely Autistic Patients

**Figure 3:**
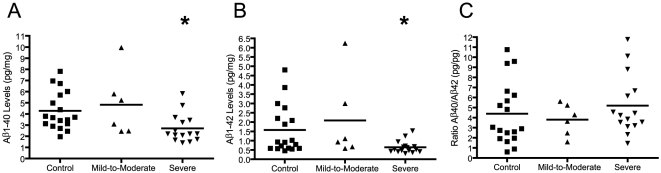
Plasma levels of Aβ1-40 and Aβ1-42 in control and autistic patients. (A) Aβ1-40 levels were quantified by a specific sandwich ELISA in plasma samples. Aβ1-40 quantities were normalized against plasma protein concentrations. Statistical significance was assessed by ANOVA. Plasma Aβ1-40 levels are significantly decreased in severely autistic patients relative to controls (*p = 0.031). (B) Aβ1-42 levels were quantified by ELISA in non-immunodepleted plasma samples. Absolute Aβ1-42 quantities were normalized against plasma protein concentrations. Statistical significance was assessed by ANOVA. Plasma Aβ1-42 levels are significantly different among all groups as analyzed by ANOVA. Differences in levels between severely autistic patients and normal controls are *nearly* significant (p = 0.055). (C) Ratios of Aβ1-40 and Aβ1-42 were calculated. No significant differences were observed between any groups (p = 0.56).

Increased plasma sAPPα and decreased sAPPβ might suggest, among other possible explanations, that full-length APP is preferentially processed along the non-amyloidogenic pathway in severe autism. To test whether production of the end-product of the amyloidogenic pathway was also affected, both Aβ1-40 and Aβ1-42 plasma levels were independently assessed by ELISA. These assays were not affected by the high protein content of plasma. Thus, unprocessed plasma samples were used for ELISA analysis. The level of Aβ1-40 was unchanged between control and mild-to-moderate autistic patients but was significantly decreased (∼60%) in severely autistic patients relative to controls following ANOVA (F = 4.487, p = 0.018) and post-hoc Dunnett's multiple comparison test (p = 0.031) (Figure 3A). Similarly, plasma Aβ1-42 levels demonstrated no change in patients with mild-to-moderate autism as compared to controls but a *nearly* significant decrease (∼50%) in severely autistic patients as compared to controls following ANOVA (F = 3.340, p = 0.047) and post-hoc Dunnett's multiple comparison test (p = 0.055) (Figure 3B). Ratios of Aβ1-40:Aβ1-42 revealed no significant difference between any groups following ANOVA (F = 0.589, p = 0.56) (Figure 3C).

**Figure 4:**
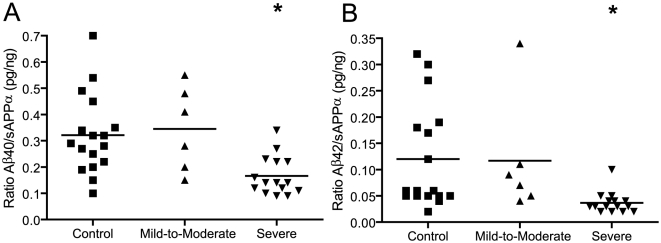
Plasma Aβ levels normalized to sAPPα. To better characterize the balance between amyloidogenic and non-amyloidogenic pathways in autism, ratios of (A) Aβ1-40:sAPPα and (B) Aβ1-42:sAPPα were calculated for each patient. Both ratios were significantly reduced following ANOVA and post-hoc Dunnett's multiple comparison test. *p = 0.003, +p = 0.012.

To further characterize changes to the balance of non-amyloidogenic and amyloidogenic APP processing products across patient samples, levels of Aβ peptides (products of amyloidogenic processing) were normalized to levels of sAPPα (product of non-amyloidogenic processing) as determined by ELISA. Levels of both Aβ1-40 and Aβ1-42 were more significantly reduced in severely autistic samples relative to controls following sAPPα normalization (Figure 4), as indicated by ANOVA [(F = 7.281, p = 0.002) and (F = 4.875, p = 0.015), respectively] and post-hoc Dunnett's multiple comparison test (p = 0.003 and p = 0.012, respectively). This highlights the inverse nature of changes to the secreted products of the amyloidogenic and non-amyloidogenic pathways in the plasma of these severely autistic patients.

### BDNF Protein Levels are Decreased in the Plasma of Severely Autistic Patients

**Figure 5:**
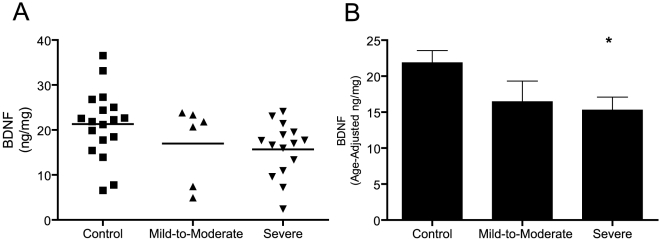
Plasma levels of BDNF in control and autistic patients. BDNF levels were quantified by ELISA in plasma samples from control and autistic patients. Absolute quantities were normalized against plasma protein concentrations. (A) Mean BDNF levels are significantly reduced in severely autistic patients compared to controls when adjusting age as a covariate and following post-hoc Sidak's adjustment for multiple comparisons. (B) Estimated marginal means for plasma BDNF levels were computed with the age covariate held at 86.18 months. The mean difference between control and severely autistic patients is enhanced when age is regressed (*p = 0.038). Thus, BDNF levels are decreased in the plasma of severely autistic patients relative to controls.

BDNF has reported links to autism and may have functional roles similar to sAPPα. To determine if the levels of BDNF were altered in the plasma of our patient set, unprocessed samples were assayed by human BDNF specific ELISA. Preliminary ELISA analyses indicated that high protein levels in unprocessed samples did not affect sensitivity and linearity of the assay (data not shown). Peripheral BDNF levels have been reported to change with age [14] so that this is an important consideration in study design. In the current study, patient age was somewhat variable but did not differ significantly between groups. In order to increase the power of analysis, we analyzed the differences in BDNF levels using an ANCOVA model with age as a covariate, disorder classification as a grouping variable, and plasma BDNF levels as a dependent variable. This analysis allowed for the variation in BDNF levels associated with disorder diagnosis to be estimated independent of variation associated with age. Plasma BDNF levels (Figure 5A) were found to be significantly decreased (∼45%; F = 3.672, p = 0.036) in severely autistic patients relative to controls following post-hoc Sidak's adjustment for multiple comparisons (p = 0.038). No significant differences were observed between mild-to-moderate autism and controls (p = 0.311) or between mild-to-moderate and severe autism (p = 0.98) as assessed by post hoc Sidak's correction. Comparing estimated marginal means (Figure 5B) derived from model parameter estimates revealed that regressing out age-associated variability enhanced the decrease in mean plasma BDNF levels observed in severely autistic patients relative to controls. No significant correlation was found between plasma levels of sAPPα and BDNF as evaluated using Pearson's correlation coefficient (R = −0.081, p = 0.634).

## Discussion

In the present report, we demonstrate that, in a much larger patient cohort, sAPPα levels are increased and BDNF levels decreased in the plasma of patients with severe autism (determined by CARS score) as compared to controls. On the contrary, sAPPβ levels are decreased in the plasma of severely autistic patients. Further, we present evidence for the first time that both Aβ1-40 and Aβ1-42 levels are significantly decreased in the plasma of patients with severe autism relative to controls. Taken together our results demonstrate that the non-amyloidogenic pathway may be preferentially active in severely autistic patients. These findings apparently do not extend to patients with mild-to-moderate autism, providing a biochemical correlate of phenotypic severity. However, it should be noted that the total number of mild-to-moderate patients was quite low in this study, likely reducing power of detecting true differences between this group and controls.

Any putative plasma-based biomarker requires replication with independent clinical cohorts. In this context, the present study replicates and extends our previous work [17], which was - to our knowledge - the first published account of abnormal levels of AD-associated biomarkers in autistic patients, thereby providing validation of this finding in two independent cohorts. The primary finding from our original study is, for the most part, in agreement with data from the current study, which uses a less restrictive classification scheme whereby severe autism is defined by CARS score >36, without requiring coexistent aggressive behavior. Specifically, our past study demonstrated that sAPPα levels were significantly increased in patients with severe autism and aggression relative to controls and mild-to-moderate autism. Our current study replicated this finding in a larger, independent cohort and, importantly, found the trend to maintain significance without the requirement for coexistent behavioral aggression. The implication is that elevated peripheral sAPPα is a general finding across the population of severely autistic patients. We also analyzed levels of various amyloidogenic products of the APP pathway. The finding that the major products of amyloidogenic processing (Aβ1-40, Aβ1-42, and sAPPβ) are all decreased in severely autistic patients lends credence to the idea that the non-amyloidogenic pathway is favored in severely autistic patients.

Some minor discrepancies between the previous and current studies exist and are described below. First, total sAPP plasma levels were found to consist of primarily the α-secretase cleaved product of APP (sAPPα) species and were elevated in severe autism with aggression in the original study. The current study did not demonstrate any significant difference between levels of total sAPP in plasma of autistic and control samples. One potential explanation is that the Western immunoblotting technique used to assay total sAPP levels in this study is only semi-quantitative and may not be sensitive enough to detect subtle differences in total sAPP between severely autistic and control patients. Second, the degree by which sAPPα was increased in the original study was much greater than in the current study. One possible explanation for this discrepancy is that, as mentioned, the criterion for inclusion into the severe autism group was less stringent for the present study as compared to the original. It is possible that plasma levels of sAPPα are increased more significantly in subgroups of autistic patients with more homogenous behavioral phenotypes and/or genotypes. The development of sophisticated classification schemes that cluster patients into groups with more phenotypic homogeneity or provide phenotype factors that approximate continuous variables for covariate analysis would likely identify larger biological differences in the levels of plasma sAPPα between groups. Such methods based on individual ADI-R items have been suggested [46], [47] and could be incorporated into future study designs. Third, the original study did not detect the decrease in plasma levels of Aβ1-40 or Aβ1-42, which were detected in the current study. However, in the original study, a trend towards decreased levels of both Aβ species was noticed despite not reaching statistical significance. The enhanced power of the current study likely enabled the true differences in Aβ peptide levels between severely autistic and control patients to attain significance.

As mentioned, the strength of the current study is in the increased power. Our previous study included 10 control and 10 autistic patient samples (3 severe with aggression and 7 mild-to-moderate). Here we include for analysis 18 control, 6 mild-to-moderate, and 15 severely autistic patient plasma samples. While the power of the mild-to-moderate analyses is still low and may explain why no differences were observed compared to controls, the power associated with control versus severe autism analyses is greatly enhanced.

Analyses from two independent laboratories have recently confirmed a portion of these findings [48], [49]. The first study demonstrated increased plasma sAPPα levels in a large sample of autistic patients relative to controls without requiring separate analysis by autism severity level. This lends further credence to our conclusion that sAPPα levels are significantly increased in the plasma of severely autistic patients. Whether this increase represents a generalized finding in autism or is restricted to a more homogeneous subgroup of autistic patients sharing a common disorder etiology is still not clear.

Autism appears to be characterized by a state of excess brain growth in some patients, as evidenced by the widely replicated finding of macrocephaly (i.e. enlarged head circumference) in 15-30% of autistic patients and up to 90% in some studies [9], [10], [12], [50]. This volumetric deviance tends to initiate around 12 months of age and continues until middle childhood after which point some patients may have smaller than average brain volumes [11], [50]. Some studies suggest an increase in the relative proportion of white matter to grey matter and cerebellar abnormalities [9], [10]. Further, a decrease in the size of corpus callosum suggests that disruptions in interhemispheric connections may exist [51]. These findings suggest that an excess of short, local circuit connections in an environment of general brain overgrowth may be favored in autism [52]. Importantly, amyloid plaque found in AD does not appear to be a component of the neuropathological description of autism [53]. Given that processing of full-length APP to sAPPα precludes the generation of Aβ, we would not expect that elevated sAPPα in autism would presage future plaque deposition. This is further supported by the decreased levels of both forms of Aβ peptide levels in the plasma of severely autistic patients tested here.

The biological activity of the secreted metabolite of APP is well-established. The first recognized biological effect of sAPP was its requirement for the normal proliferation of fibroblasts in culture [54] in addition to the promotion of neuronal PC12 cell adhesion to culture substratum [55]. Studies on cultured neurons and animals quickly followed and have since identified trophic effects that include the: 1) enhancement of neurite outgrowth [56], [57], [58], [59], [60], [61], 2) stimulation of neural stem cell proliferation [62], [63], [64], differentiation and migration [65], 3) promotion of synaptogenesis [66], [67], [68], and 4) modulation of synaptic plasticity, learning and memory [69], [70]. Additionally, sAPP has been demonstrated to protect neurons against a variety of insults that include glucose deprivation and excitotoxicity, Aβ and reactive oxygen species, and ischemic brain injury [71], [72], [73]. Following the discovery of multiple secretase activities [74], [75], sAPPα was found to mediate the majority of neuronal-enhancing effects. Specifically, the neuroprotective action of sAPPα against glucose deprivation, excitotoxicity, and Aβ toxicity is 100-fold greater than that mediated by sAPPβ [76]. The effects of excess sAPPα are unknown and only beginning to be studied. Recently, Conti et al. [77] found plasma sAPPα higher in a small sample of adults with Down's syndrome compared to both intellectually disabled and control subjects. Autism has been associated with 10% of children with Down's syndrome.

Based on the data from our past and current work, we hypothesize that the elevated levels of peripheral sAPPα observed in severe autism is an indicant of metabolic imbalance in the CNS – specifically an anabolic state driven by high levels of sAPPα and perhaps other neurotrophic factors (other than BDNF) in the brains of these patients. In our hypothesis, this state of anabolic excess is likely to contribute to the biological substrate of the disorder. Given the wide array of neurotrophic effects, we predict that excessive levels of sAPPα in the brains of severely autistic patients would induce neurodevelopmental changes that mimic the volumetric and neuropathological changes observed in autism [11]. What may underlie the increase in sAPPα in autism? A decrease in beta-secretase processing is the most likely possibility, supported by the decrease in levels of sAPPβ and both Aβ1-40 and Aβ1-42 peptides observed in this study. An increase in alpha-secretase processing is a second, not mutually-exclusive, possibility.

We also cannot rule out changes to holo-APP expression. In this context, mGluR5-dependent signaling may contribute. A mechanism for mGluR5-dependent translation of APP recently has been described [78]. In this pathway, translation of APP mRNA is constitutively inhibited by binding of (Fragile X mental retardation protein) FMRP to the mRNA. FMRP is a RNA-binding protein that represses translation of targeted mRNA transcripts, including APP [78], locally within dendrites [79] and in an activity-dependent manner. A proposed mechanism underlying repression of APP translation by FMRP involves recruitment of CYFIP1, a FMRP-binding partner, to the APP transcript. CYFIP1 then binds to eIF4E and inhibits its interactions with other binding partners of the translation initiation complex [80]. Following activation of mGluR5 receptors, FMRP dissociates from the APP mRNA and translation proceeds. Interestingly, excessive mGluR5 signaling events (due to absence of FMRP) have been described in Fragile X syndrome, a syndrome with high prevalence of co-morbid autism [81], [82]. Genetic down-regulation of mGluR5 signaling was shown to reverse the behavioral deficits in FMRP-KO mice [83], [84]. If mGluR5 signaling is enhanced in severely autistic patients, excessive APP translation would be expected, inevitably leading to higher sAPPα levels. Simple mGluR5 antagonism would be expected to reverse this effect.

A separate candidate neurotrophin shown to be dysregulated in the blood and serum of autistic patients is BDNF. The original report on peripheral BDNF levels found a significant increase in archived neonatal dried blood samples from autistic patients relative to controls [16]. This led to the hypothesis that elevated BDNF in the CNS may contribute to brain overgrowth observed in autism [85]. Specifically, an early pre-or postnatal rise in BDNF may herald eventual brain overgrowth while other factors may support overgrowth at later ages. Follow-up studies, including some from independent labs have either demonstrated a similar increase in peripheral BDNF with autism [14], [15], [86], found no association with autism [87], or demonstrated reduced peripheral BDNF levels with autism [88], [89] , as found in this report. The discrepancy between studies likely arises from differences in sample types analyzed, assay methods used, repeated freeze-thaw cycles and age of patients when samples were collected. In typically developing individuals, peripheral BDNF levels have been found to rise rapidly with age [14], and then decrease into adulthood [89]. Consequently, due to the anticipated effect of age on BDNF, and because of a somewhat high variation of age in our tested samples, we utilized patient age as a covariate when analyzing BDNF levels. Using the ANCOVA model, we observed a significant decrease in the levels of plasma BDNF in severely autistic patients relative to controls. If peripheral BDNF levels correlate well with brain BDNF levels as has been suggested [90], our finding of decreased plasma BDNF would suggest that BDNF alone cannot account for brain overgrowth in autism. Indeed, a recent study has found decreased BDNF levels in the brains of autistic patients as compared to controls and has suggested this may lead to reduced BDNF-Akt-Bcl2 anti-apoptotic signaling in autism [91]. This finding lends further credence to the idea that sAPPα or other neurotrophic factors must contribute to this phenotype. At the neuronal and synapse level, it is noteworthy that BDNF-p75NTR-dependent signaling appears to promote developmental axon pruning through axon degeneration [92]. A CNS environment of excess sAPPα and reduced BDNF might be expected to promote neuronal proliferation, differentiation, and synaptogenesis along with a deficit in axon pruning, leading to a state of general brain overgrowth.

Neurobiologically, the contrasts between neurodegenerative conditions (such as Alzheimer's and some forms of schizophrenia) and conditions involving overgrowth/overactivity of growth pathways (such as FXS and autism) are quite intriguing and our recent work has addressed the molecular-pathway underpinnings of such contrasts (Sokol et al, 2011). The present work should lay the path for further research beyond autism. For example, it is not clear whether the sAPPα-mediated brain overgrowth pathway described herein is applicable to autism *only* or can be extended to other psychiatric disorders with either brain overgrowth or undergrowth. Autism-spectrum and psychotic-spectrum conditions (i.e. schizophrenia, bipolar disorder, and major depression) represent two major suites of disorders of human cognition and behavior with altered development and function of the social brain [93], Should one then expect sAPPα to follow different trajectories in these conditions? What about the gender bias in autism, where the prevalence in boys is 1 in 70, as opposed to 1 in 110 for all children? Could this be explained by “genomic conflict” during brain development [94], or is it due to some unknown environmental or epigenetic factors? [95]. Future studies will need to carefully investigate levels of sAPPα, BDNF and other protein markers in samples from different genders and other psychiatric conditions to answer some of these questions.

In summary, we have confirmed the finding from our original report of increased sAPPα in the plasma of severely autistic patients. We have also found that this elevation is not limited to only severely autistic patients exhibiting aggressive behaviors. Further, we demonstrate for the first time that Aβ1-40, Aβ1-42 and sAPPβ levels are reduced in the plasma of severely autistic patients. These biochemical changes may be characteristic of a subgroup of patients with a more homogenous phenotype and as such could be a useful biomarker to track in future studies or perhaps for inclusion in a future diagnostic panel of biomarkers. Furthermore, we predict that the peripheral elevation of sAPPα is indicative of high CNS sAPPα levels and that this contributes to an anabolic state that underlies macrocephaly, brain overgrowth and other neurodevelopmental abnormalities in autism. Given that the highest rate of brain overgrowth in autism appears to occur in the first few years of life [50], measurement of peripheral sAPPα levels during this time of development could reveal even greater differences between severely affected patients and controls, and serve as a marker of autism severity. It must be stressed that it is presently unknown if the change in peripheral sAPPα is mirrored in the brain of severely autistic patients. These samples are difficult to obtain due to low age of the required control and affected patients. Nevertheless, future studies are required to address this caveat. Taken together, this research should have a significant impact, not only in clarifying the etiology of autism and related neurodevelopmental disorders, but also in guiding future research on the sAPP and mGluR pathways at the cellular level and as putative therapeutic drug targets.

## Materials and Methods

### Ethics statement

This study was approved by the Indiana University School of Medicine Institutional Review Board's Human Subjects Committee (IUPUI campus, Indianapolis, IN, USA). All parents signed informed consent for their child's participation. Additionally, all research was conducted according to the principles expressed in the Declaration of Helsinki.

### Participants, diagnostic inclusion criteria, and measures

The inclusion criteria for the autism group were a diagnosis of Autistic Disorder based on the DSM-IV criteria determined by an experienced neurologist (DKS), which was further corroborated by the Autism Diagnostic Interview-Revised (ADI-R) [37]. The ADI-R is a comprehensive, semi-structured parent interview that assesses a child's developmental history and relevant behaviors characteristic of autism and generates a diagnostic algorithm for Autistic Disorder. Children with genetic causes of autism, e.g., Fragile X syndrome, were excluded. Typically developing age- and gender-matched volunteers comprised the control group. These children had no major medical problems, were on no medication, and had met their developmental milestones and/or were functioning in typically developing classrooms. The Childhood Autism Rating Scale (CARS) [38] was obtained for all participants. This parent questionnaire is a 15-item behavioral rating scale developed as a screen for autism and to classify severity. Validity and reliability for the CARS have been established [38]. Participants were administered age appropriate cognitive testing (i.e., Mullens Scales of Early Learning [39] or Wechsler Intelligence Scale for Children-IV [40]).

### Plasma analyses

#### A. Removal of albumin from human plasma

For some antibody-dependent assays described below, the levels of highly abundant proteins present in plasma samples interfered with assay sensitivity, specificity and/or linearity due to non-specific protein interactions. To circumvent these issues, aliquots of raw plasma samples were pre-fractionated by depletion of the majority of human plasma albumin using ProteomeLab IgY human serum albumin (HSA) spin column-based proteome partitioning kit (Phenomenex, Torrance, CA). Briefly, plasma samples were pre-filtered using Corning Costar Spin-X 0.45 µm cellulose acetate filters, diluted 25-fold using kit-supplied 1x dilution buffer, and then incubated with anti-HSA-coated beads for 15 minutes. Albumin-depleted plasma was collected as flow-through. Preliminary SDS-PAGE experiments utilizing flow-through, wash and eluate fractions demonstrated that a large majority of albumin was successfully removed by this procedure (data not shown). These diluted, albumin-immunosubtracted samples were then used directly without further concentration for biochemical analyses as indicated. Concentration was not performed in order to avoid variation in the final volume of HSA-immunosubtracted plasma obtained after concentration.

#### B. Measurement of total protein content in the plasma

Two microliters of raw plasma were diluted ten-fold with freshly prepared Dulbecco's phosphate buffered saline (DPBS) and measurement of total protein content in the plasma was performed using Bradford protein assay method as described previously [41]. Total protein content of all HSA-immunosubtracted plasma samples was also determined using the Bradford method.

#### C. Levels of total sAPP by Western immunoblot assay

To avoid non-specific binding of antibody with abundant HSA in raw plasma, we used HSA-immunosubtracted plasma when assaying levels of total sAPP by Western immunoblotting. Briefly, equal volume of denatured HSA-immunosubtracted plasma was loaded on two 26-lane Bis-Tris Criterion XT gels (BioRad) and electrophoresis was carried out for 1 hour at 180 V and the proteins from the gels were electrophoretically transferred onto a PVDF membrane and blocked in 5% non-fat milk as previously described [42]. After blocking, the membrane was probed with monoclonal anti-APP antibody (Clone 22C11, Millipore, MA, USA), which recognizes the 66-81 amino acid sequence of APP (N-terminus). This epitope is present in all sAPP forms (such as sAPPα, sAPPβ and sAPP γ) and their post-translational modifications and, thus, allows detection of the *total* sAPP pool. After incubation with HRP-conjugated anti-mouse secondary IgG, blot signals were obtained using the ECL method. Band densities were quantified using Image J software [43] and densities were normalized to total protein content of HSA-immunosubtracted plasma.

#### D. Levels of sAPPα by ELISA

For all ELISAs, we performed initial experiments with different volumes of plasma samples to establish linearity of the assays. Equal volumes of HSA-immunosubtracted plasma were used to assay the levels of sAPPα by the sandwich ELISA method (Immuno-Biological Laboratories, Gumma, Japan or IBL). Briefly, an equal volume (50 µl) of HSA-immunosubtracted plasma was added onto wells pre-coated with capture monoclonal anti-human sAPPα antibody (clone 2B3) and incubated overnight at 4°C. After washing several times, the wells were incubated with HRP-conjugated monoclonal anti-APP detection antibody (clone 10D1). Colorimetric signals were obtained after addition of chromogen substrate followed by application of 1N sulfuric acid. A standard curve was prepared by using known amounts of recombinant human sAPPα protein. sAPPα values (ng/ml) in all HSA-immunosubtracted plasma samples were normalized to total protein content of HSA-immunosubtracted plasma.

#### E. Levels of sAPPβ by ELISA

We have measured the levels of sAPPβ by a commercially available ELISA kit (IBL) as per the manufacturer's protocol. Briefly, 50 µl of HSA-immunosubtracted plasma from each control and autism subject was separately loaded onto a prelabeled well of a 96-well ELISA plate and allowed to incubate overnight at 4°C with pre-coated anti-human sAPPβ wild type antibody, which recognizes C-terminus of human sAPPβ. After several washes, the wells were incubated with HRP-conjugated anti-APP detection antibody (10D1). Colorimetric signals were obtained in the same way as mentioned in the section of sAPPα ELISA. A series of known amounts of human recombinant sAPPβ samples were likewise incubated and processed to prepare a standard curve. Similar to other ELISAs used in this report, sAPPβ values were normalized by the total protein content of HSA-immunosubtracted plasma.

#### F. Levels of Aβ1-40 peptide by specific ELISA

Aβ1-40 levels were measured using an Aβ-40-specific sandwich ELISA kit (Biosource, MN, USA) as described previously [44]. Wells were pre-coated with monoclonal antibody specific for the N-terminal amino acid sequence of human Aβ peptide. Equal volumes of raw plasma samples mixed with detection antibody (rabbit polyclonal antibody specific for C-terminus of human Aβ1-40) were incubated at room temperature for three hours. Colorimetric signals were obtained in a similar fashion to other ELISAs [44]. A standard curve using known amounts of Aβ1-40 was also performed and plasma Aβ1-40 values (pg/ml) were normalized to total protein content of raw plasma samples.

#### G. Levels of Aβ 1–42 peptide by specific ELISA

Aβ1-42 levels were measured using an Aβ-42-specific ELISA kit (Biosource, MN, USA). Wells were pre-coated with monoclonal anti-Aβ antibody which is specific to the N-terminal sequence of human Aβ peptide. The detection antibody was specific for the C-terminal sequence of human Aβ1-42 peptide. The assay was performed as per the manufacturer's guideline. Levels of Aβ1-42 in the plasma were calculated from a standard curve prepared using known amounts of human Aβ1-42 peptide and normalized to the total protein content of raw plasma samples. Ratios of Aβ1-40 and Aβ1-42 were calculated from total protein content-normalized values.

#### H. Measurement of BDNF in plasma by ELISA

For BDNF assay, equal volume of platelet-free raw plasma was loaded into a plate pre-coated with mouse monoclonal anti-BDNF antibody (R & D Systems, MN, USA) and incubated at room temperature for two hours. HRP-conjugated monoclonal anti-BDNF detection antibody was added to the wells and further incubated for one hour. After brief wash, substrate solution was applied to the wells and incubated for 30 minutes. The reaction was stopped by application of Stop Solution (2N sulfuric acid). The colorimetric signals were measured using a microplate reader (BioRad, CA, USA). A standard curve with known amounts of recombinant human BDNF was also performed to calculate absolute quantities of BDNF present in each plasma sample. This BDNF value was normalized to the total protein content of each plasma sample, as measured by the Bradford method.

### Data Analysis

All statistical analyses were performed using SPSS Statistics 17.0 software and all plots were constructed using GraphPad Prism 4.0 software. Unless otherwise stated, all data are presented as mean ± SEM. Further, ANOVA or ANCOVA analyses were performed where indicated under the framework of the general linear model. Post-hoc multiple comparisons were assessed using either Dunnett's t-test or Sidak's multiple comparison test, as indicated. The α threshold was set to 0.05 for determining statistical significance in all cases.
